# Regulation of Symbiotic Nitrogen Fixation in Legume Root Nodules

**DOI:** 10.3390/plants8090333

**Published:** 2019-09-06

**Authors:** Andrés R. Schwember, Joachim Schulze, Alejandro del Pozo, Ricardo A. Cabeza

**Affiliations:** 1 Departamento de Ciencias Vegetales, Facultad de Agronomía e Ingeniería Forestal, Pontificia Universidad Católica de Chile, Santiago 306-22, Chile; 2 Department of Crop Science, Section for Plant Nutrition and Crop Physiology, Faculty of Agriculture, University of Goettingen, Carl-Sprengel-Weg 1, 37075 Goettingen, Germany; 3 Centro de Mejoramiento Genético y Fenómica Vegetal, Facultad de Ciencias Agrarias, Universidad de Talca, Talca 3460000, Chile; 4 Departamento de Producción Agrícola, Facultad de Ciencias Agrarias, Universidad de Talca, Campus Talca, Talca 3460000, Chile

**Keywords:** nitrogen fixation regulation, legume nodule, carbon metabolism, nitrogen metabolism, oxygen supply

## Abstract

In most legume nodules, the di-nitrogen (N_2_)-fixing rhizobia are present as organelle-like structures inside their root host cells. Many processes operate and interact within the symbiotic relationship between plants and nodules, including nitrogen (N)/carbon (C) metabolisms, oxygen flow through nodules, oxidative stress, and phosphorous (P) levels. These processes, which influence the regulation of N_2_ fixation and are finely tuned on a whole-plant basis, are extensively reviewed in this paper. The carbonic anhydrase (CA)-phosphoenolpyruvate carboxylase (PEPC)-malate dehydrogenase (MDH) is a key pathway inside nodules involved in this regulation, and malate seems to play a crucial role in many aspects of symbiotic N_2_ fixation control. How legumes specifically sense N-status and how this stimulates all of the regulatory factors are key issues for understanding N_2_ fixation regulation on a whole-plant basis. This must be thoroughly studied in the future since there is no unifying theory that explains all of the aspects involved in regulating N_2_ fixation rates to date. Finally, high-throughput functional genomics and molecular tools (i.e., miRNAs) are currently very valuable for the identification of many regulatory elements that are good candidates for accurately dissecting the particular N_2_ fixation control mechanisms associated with physiological responses to abiotic stresses. In combination with existing information, utilizing these abundant genetic molecular tools will enable us to identify the specific mechanisms underlying the regulation of N_2_ fixation.

## 1. Introduction

Legume crops have economic relevance because they are consumed by millions of people around the world, as well as having outstanding nutritional properties. Legume seeds provide proteins (double or triple most cereals), dietary fibers, and carbohydrates, as well as fatty acids, folic acid, vitamins, and minerals, among others, which are beneficial to human health [1]. The world’s cultivated surfaces of pulses and soybeans are 82.4 and 121.5 million ha, respectively, and the production is 82 and 335 million tons, respectively [2].

The symbiotic relationship between soil bacteria, collectively known as rhizobia (which includes the genera *Rhizobium*, *Bradyrhizobium*, *Mesorhizobium*, and *Sinorhizobium*), and legume roots, generates nodules (a new differentiated organ), which fix atmospheric di-nitrogen (N_2_) through the action of the nitrogenase enzyme [3]. The specific association between the nodulating root and its hosting plant is mainly controlled by the exchange of two different compounds: nitrogen (N) and carbon (C). The plant supplies reduced-C (carbohydrates) to the bacteria, which are used as food and energy and to stimulate the N_2_ fixation process, while the nodules return reduced-N to the plant [4]. It is estimated that a total of 50–200 MT of N are biologically fixed in agricultural systems annually, greatly contributing to the productivity of legume and non-legume crops (grown in association or in rotation with legumes), as well as to the global N cycle [5]. This biological N_2_ fixation diminishes both the risks of pollution caused by intensive synthetic N fertilizer use and production costs [6].

Although most rhizobia-legume symbiotic association research has focused on rhizobial infection and nodule initiation/formation processes (e.g., see the reviews by Poole et al. [7] and Buhian and Bensmihen [8]), relatively less attention has been paid to the regulatory aspects of symbiotic N_2_ fixation. In this context, most of the studies about the controlling aspects of symbiotic N_2_ fixation deal with N-metabolism [9] and C-metabolism [10], the oxygen flux in/out of the nodule [11], oxidative stress on N_2_ fixation [12], and N_2_ fixation under stressful environments [13,14,15], among other factors.

Consequently, the objectives of this paper were to examine and discuss the main advances in the regulation of symbiotic N_2_ fixation of legumes. We focused special attention on the metabolic and molecular aspects involved in this process, particularly encompassing the proteins and carbohydrates that play a role in this complex regulatory network.

## 2. Overview of the Control of the Legumes´ Symbiotic Nitrogen Fixation

The symbiotic nitrogen-fixing rhizobia associated with legumes include 14 genera and more than 98 species [16]. One essential aspect that differentiates rhizobial species is their growth rate, exhibiting either fast or slow growth, which is associated with the synthesis of acidic N and alkaline N compounds, respectively [17]. Nodules also differ in their shape, which can be determinate (spherical with lenticels and a synthesis of ureide compounds) or indeterminate (cylindrical and branched, with a synthesis of amide compounds). Examples of determinate nodules are observed in soybeans, common beans, and other species grown in tropical and sub-tropical areas, whereas peas, alfalfa, and clover produced in temperate regions display indeterminate nodules [18]. Consequently, different regulatory principles among various symbiotic systems or a chain of regulatory events involving several mechanisms may exist, rather than an all-embracing regulatory mechanism of N_2_ fixation [19].

To optimize a plant’s N-demand with its nodule activity, several regulatory processes have been specifically developed by this symbiotic relationship: (i) N- and C-metabolism controlled by several enzymatic pathways; (ii) controlled O_2_ supply to nodules by leghemoglobin (Lb) and a restriction of O_2_ diffusion by a physical barrier, the oxygen diffusion barrier (ODB); (iii) the production of reactive oxygen species (ROS) and reactive nitrogen species (RNS); and (iv) molecular control by adjustment of the nodule number and N_2_ activity. These processes have complex control mechanisms, including regulation of the gene expression network and the nutrient-dependent cellular metabolism, which takes place in the shoot of the legume plant via sensing and long-distance signaling cross-talk [20].

## 3. Nitrogen-Metabolism

Legumes contain higher levels of N in leaves and shoots, but lower rates of photosynthesis and plant growth per unit of N in the plant compared to cereals [21,22]. N is supplied by symbiotic fixation, but also from the soil, in proportions depending on nodule activity and soil N availability [23]. Plant growth and N content are increased with an external N supply in both legume and non-legume plants, but the dependence on this supply is stronger in non-legumes [24]. The increase in any form of N in soil or nutrient solution reduces the number of root nodules and N_2_ fixation rates [25,26].

Several experimental approaches have provided evidence that the overall plant N status regulates nodule activity and nitrogen fixation rates [27,28]. Some models have also supported the positive correlation between plant N-demand and nitrogenase activity [28,29]. The N-status of plants is most likely detected in the shoot, where specific signals could be transmitted to the nodulated roots encompassing a message regarding the whole-plant N-status [30,31]. The dependence of N_2_ fixation rates on the N-status of the plant has been supported by the following evidence [19]: (a) N_2_ fixation rates were at the maximum at pod filling when N was at its highest demand [32]; (b) the nitrogenase activity decreased when the remobilization of N from senescence leaves increased [33]; and (c) N_2_ fixation rates varied, depending on the phenological stage of plants [28,34].

The pool of amino-N compounds that is continuously cycled between roots and shoots has been studied over the past decades [9]. In the past 25 years, 13 different types of small (5–75 amino acids (AAs)) signaling peptide species have been identified and shown to control several developmental processes in plants [35], which were previously assumed to be controlled exclusively by phytohormones [36]. As major constituents of the phloem circuit, AAs seem to be one of the main candidates playing a role as long distance signals that are capable of conveying information to the nodule about the shoot N-status [20]. This concept has been supported by the promoting effect on N_2_ fixation rates of γ-aminobutyric acid (GABA) [37,38] and the inhibitory effect of asparagine (ASN) [39], when they were exogenously supplied to the phloem of nodulated *Medicago truncatula*. Based on the literature, different N-compounds and enzymes have been proven to be involved in the control of N_2_ fixation rates, such as glutamate (GLU) [33,40], glutamine (GLN) [41], glutamine synthetase (GS) [42,43], aspartate (ASP) [44], ASN [39], ureides [45], polyamines [46], and proline (PRO) [47], among others. Despite the large amount of indirect evidence for N-metabolism regulation of N_2_ fixation rates, no equivocal proof for one particular compound or specific related mechanism controlling nodule N_2_ fixation has been identified to date.

Other components have been identified as important for the regulation of N_2_ fixation that are related to N-metabolism, such as transporters for N exported out of nodules, like ureid permeases (UPS1) in *Phaseoulus vulgaris* [48]. More recently, using the miRNA interference technique, the amino acid permease (AAP6) expressed in pea nodules was identified as being responsible for retrieving organic N from the apoplasm and transporting it into the symplasm of cells near the vascular bundle for phloem-xylem loading [49]. This indicates that the apoplastic pathway is key for amino acid movement to vascular bundles and that AAP6 is essential to bypassing the barrier imposed by the Casparian strip of vascular tissues in pea nodules. Down-regulation of AAP6 in pea nodules resulted in a defective export of reduced N, N accumulation in nodules, a low N content in shoots, and N_2_ fixation stimulation, suggesting that the N status in leaves probably induced a phloem-mobile N deficiency signal [49].

## 4. Carbon-Metabolism

The amounts of N_2_ fixed by pulses and forage legumes in different environments have been reported to be highly correlated with shoot biomass, indicating that about 20–22 kg shoot N ha^−1^ are fixed for every ton of shoot dry matter accumulated [5,50,51,52]. This suggests that the amount of N_2_ fixed by legumes is regulated by plant growth and dry matter production. Symbiotic N_2_ fixation consumes considerable energy, and thus requires a large amount of assimilates [19]. There is evidence that legumes use more photosynthates for N assimilation if N comes from N_2_ fixation, compared to N uptake from the soil [53]. The C cost per unit of fixed N (g C per g N fixed) varies widely with species, growth stage, and environmental conditions, and ranges from 1.4 to 12 g C per gram fixed-N [54]. Considering 2 mg of respired C (mg fixed N)^−1^, the C respired for driving N_2_ reduction corresponds to around 25% and 176% of C used for shoot and root growth, respectively. Consequently, particularly under stressful conditions, legume plants closely control N_2_ fixation reactions to avoid the exhaustion of plant carbohydrate reservoirs [20,55].

It has been proposed that under non-stressful conditions (i.e., an optimal photoperiod and light intensity, and non-limited water and nutrient availability), the current photosynthesis or assimilate supply to nodules does not regulate N_2_ fixation activity [19,56]. This is supported by the fact that nodules have been observed to accumulate starch, indicating a sufficient or excessive C supply [57]. In addition, exposing plants under optimal light conditions to their CO_2_ compensation point did not affect their nitrogenase activity for as long as 6 h [58]. In addition, N_2_ fixation appears to continue without interruptions to normal day/night cycles [34]. However, a number of experiments have shown that plant biomass and N_2_ fixation increased at elevated CO_2_, compared to ambient CO_2_, indicating that greater photosynthate availability stimulates N_2_ fixation [59,60,61,62]. The increase in N_2_ fixation under elevated CO_2_ has been associated with a greater nodule number and mass, and total nitrogenase activity [60].

**Figure 1:**
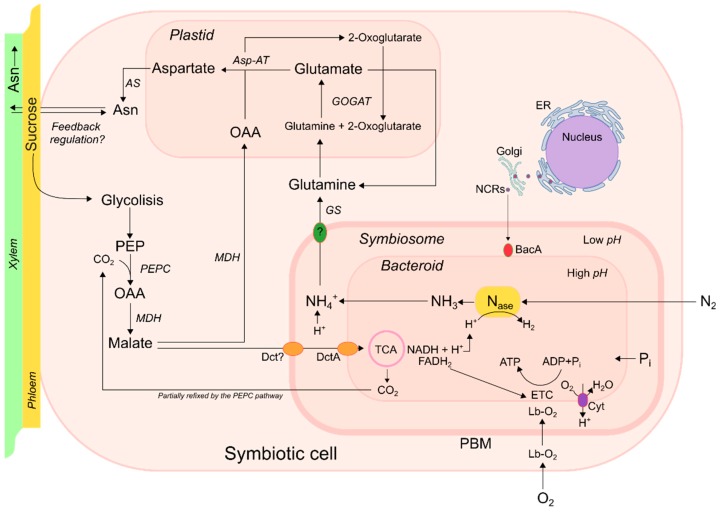
Infected cell with a differentiated bacteroid in an indeterminate nodule. Nitrogenase reduces di-nitrogen (N_2_) into ammonia (NH_3_) in the inner space of the bacteroid (high pH), which is then protonated into ammonium (NH_4_^+^) in the symbiosome space (low pH). Leghemoglobin (Lb) transports O_2_ to the bacteroid cytochrome (Cyt) of the electron transport chain (ETC). Sucrose is downloaded in the cytoplasm, where it is transformed by glycolysis pathways (PEPC-MDH) into malate, the main source of carbon skeletons for N transport out of the nodule and reductant power for driving N_2_ fixation. Malate is transported by dicarboxylate transport (Dct and DctA), either placed in the peribacteroid membrane (PBM) or the bacteroid membrane. A cation channel permeable to NH_4_^+^ has been proposed as exporting NH_4_^+^ across the PBM [69]. NH_4_^+^ is transformed into asparagine (Asn) through the GS-GOGAT pathway using oxaloacetate (OAA) as a substrate. Asn is most likely involved in a negative feedback regulation of N_2_ fixation. Part of the CO_2_ from the tricarboxylic acid (TCA) cycle is recycled by the phosphoenolpyruvate carboxylase (PEPC). Nodule-specific cysteine-rich (NCR) peptides drive the final transformation of bacteria into a bacteroid by a recognition protein system (BacA) located in the bacteroid membrane. Figure adapted from: Fischinger [70], Oldroyd et al. [67], Udvardi and Poole [63], and Sulieman and Tran [20].

An idealized bacteroid in an indeterminate nodule is shown in Figure 1. Photosynthetic sucrose is exported through the phloem and unloaded into the nodule cortex, where it is metabolized into malate by the glycolysis pathway to phosphoenolpyruvate (PEP), which is ultimately reduced to malate, which is the primary fuel for N_2_ reduction into ammonia in the bacteroid [63]. Sucrose is transported via membrane transporters, such as MtSWEETT11, to uninfected cells, to be broken down into malate [64]. The symbiosome membrane has a dicarboxylic transporter which is able to carry malate to the bacteroid [63,65]; other malate transporters are most likely involved in the process, such as the aluminum-activated malate transporter (ALMT) family, which may play a role in transporting organic acid via nodule vasculatures to the bacteroid [66]. There is abundant evidence indicating that malate is the principal source of energy provision for the bacteroid [63,67] since it accumulates at very high concentrations in effective symbiotic nodules. In addition, malate is easily transformed into oxaloacetate (OAA) through the activity of malate dehydrogenase (MDH). Furthermore, OAA, through the GS-GOGAT pathway (Figure 1), serves as a C skeleton for the formation of asparagine (ASN), which acts as the principal N export compound from the nodule in temperate legumes [39]. In the case of determinate nodules, the principal N-compounds exported from the nodules are ureides, but C and N metabolism is very similar [68].

Since sucrose via glycolysis is the main source of reduced C for malate production, its supply is essential for the synthesis of this organic acid. However, sucrose could also be hydrolyzed in vascular tissues and the hexoses produced are transported to the center of the nodule’s active zone to form starch [71]. Using developmental, transcriptional, and metabolic approaches, sucrose phosphate synthase (SPS) has been proven to be the enzyme responsible for sucrose synthesis in plants and its activity plays a crucial role in nodulated legumes, such as *Medicago truncatula* [72]. High activity in nodules suggests that SPS synthesizes sucrose from the starch breakdown [71]. Expression analysis in leaves and nodules of *Medicago sativa* showed that an enhanced isoform of SPS (MsSPSA) is present in nodules, where it acts over three times faster than in leaves, based upon its activity (*V_max_*) in both tissues [71]. The overexpression of SPS in nodules of *M. sativa* transgenic plants positively affected the number of nodules, the amount of asparagine and glutamine synthesized, and the exportation of nitrogen fixed from the nodule, demonstrating that augmented SPS activity enhances the plant performance and N status, which indicates that the presence of C from sucrose improves N uptake in symbiotic nodules [73].

The carbonic anhydrase (CA)-phosphoenolpyruvate carboxylase (PEPC)-MDH is a key pathway for C feeding and C skeleton provision for N assimilation of the bacteroid [32]. Warembourg and Roumet [74] concluded that nearly 55% of C respired for N_2_ fixation was re-fixed via PEPC, of which 25% was used for N assimilation, while the rest again underwent immediate respiratory conversion into CO_2_ (Figure 1). The importance of the CA-PEPC-MDH pathway is also evident based on the analyses of enzymes involved in this metabolic route; CA activity has been proven to be high in nodules [75], especially in cortical cell layers at supra-ambient oxygen concentrations around nodules [76]. The PEPC concentration in *Medicago sativa* nodules has been observed to be high and comparable to that of young maize leaves, based on their molecular weight similarity, resulting in increased PEPC activity in active nodules [77]. Additionally, the PEPC gene expression and its activity rates appear to be closely related to N_2_ fixation rates and a decrease of its expression, due to an antisense strategy, which resulted in impaired N_2_ fixation [78]. Furthermore, increasing the CO_2_ concentration around alfalfa nodules resulted in higher CO_2_ fixation via PEPC-MDH, coinciding with a higher N_2_ fixation rate/capacity and plant growth compared to plants with normal CO_2_ concentrations [32]. The MDH activity has also been observed to be very high in legume nodules [79], especially one specific nodule-enhanced MDH form (neMDH) in alfalfa that possesses unique kinetic properties, which strongly favor the OAA to malate reaction [10,80], leading C metabolism towards the production of malate. Similar nodule-enhanced forms of MDH have been found in soybean and pea studies [81]. The over-expression of neMDH in alfalfa resulted in more efficient N_2_ fixation and increased N_2_ fixation rates [82], which suggests that increasing assimilate conversion into malate might be a feasible strategy for improving N_2_ fixation. Molecular approaches have unequivocally shown that neMDH mRNAs are more abundant in infected cells of active nodules, while mRNA of cytosolic MDH isoforms (cMDH) is present in uninfected cortical cells, and is probably involved in O_2_ permeability [10]. Under P deficiency, cMDH showed enhanced activity in *Lupinus angustifolius* nodules, indicating its contribution of malate to maintain N_2_ fixation, which is most likely the consequence of low O_2_ concentrations in the inner cell of the nodule produced by a reduced gas permeability [83].

## 5. Nodule Oxygen Supply

Oxygen (O_2_) supply to the nodule´s interior is a key factor in the regulation of nodule activity [84]. While a decreased O_2_ concentration around nodules might prevent N_2_ fixation through a limited O_2_ supply for ATP production, an increased O_2_ concentration around the nodule might augment the risk of nitrogenase destruction [85]. However, exhaustive measurements have shown that changes in O_2_ concentrations around the nodules cause an adjustment in the nitrogenase activity due to a relaxation of the gas diffusion at low O_2_ partial pressure and an increase in the resistance if the O_2_ concentration is high [86]. An adjustment in O_2_ diffusion has been postulated since step-wise increments of O_2_ concentration have produced a temporal nitrogenase inhibition, followed by a recovery to previous activity [86]. Since a high O_2_ concentration around nodules transiently increases nitrogenase activity, it has been hypothesized that nitrogenase is O_2_-limited. The transient behavior of nitrogenase activity that increases O_2_ could be the result of refined nodule adaptations, which are mainly associated with physical [87], metabolic/morphological [88,89], osmoregulatory functioning [90], and molecular [91] mechanisms.

Very precise measurements have shown sharp declines in O_2_ concentrations between the nodule cortical and inner cells [92]. Therefore, a physical barrier has been proposed to control the diffusion of O_2_ to the interior of the nodule active zone, the so-called oxygen diffusion barrier (ODB). The existence of the ODB has been supported by the fact that a concomitant accumulation of H_2_ gas (product of H^+^ reduction by nitrogenase) inside the nodule is accompanied by a reduction in the O_2_ concentration [93,94]. These observations are consistent with a variable gas diffusion barrier that controls the entrance and exit of gases. Morphological changes and the metabolite accumulation of uninfected cells have been proposed to be the main mechanisms involved in controlling the ODB [88,89]. The accumulation of cytosolic MDH in uninfected cells may cause size changes through shrinking and swelling, like stomata cells of leaves [10,89]. Moreover, membrane depolarization of cortical cells has been measured after exposition to a high O_2_ concentration [90]. In this model, membrane depolarization allowed fluxes of inorganic ions that controlled the cell turgor, as in stomata cells, therefore controlling gas diffusion. Phytase activity may also be involved in O_2_ diffusion, especially under P deficient conditions, as has been studied using inbred lines of *Phaseolus vulgaris* [95]. On the basis of molecular analyses, Avenhaus et al. [91] concluded that the inhibition of nitrogenase activity after sudden increases of O_2_ exposure was counteracted rapidly, until the pre-treatment level was reached by neo-formation of the enzyme. This reaction could be induced by an increased formation of nodule-specific cysteine-rich (NCR) peptides, requiring an efficient iron supply to the bacteroid, which is most likely mediated by nicotianamine [91]. Overall, several experimental works have shown that the ODB plays an important role in regulating O_2_ diffusion, especially when plant growth is restricted (i.e., mineral nutrient deficiencies). In this case, a down-regulation of N_2_ fixation is required to meet the N demand of the legume plant [96].

Nodules’ high respiration rates are maintained by the effective transport of O_2_ to the bacteroid through the protein leghemoglobin (Lb), which is fundamental to maintaining a high ATP production rate (Figure 1) [97]. The existence of Lb in nodules is a prerequisite for N_2_ fixation since it buffers the free O_2_ concentration to micro- and nano-mole ranges [97,98]. In *Lotus japonicus*, five genes encode for Lb, of which three are exclusively expressed in nodules and indispensable for successful N_2_ fixation [99]. Interference in the expression of these symbiotic Lb genes in *L. japonicus* caused an increase in O_2_ in the infected zone, resulting in a lower O_2_ buffer capacity [97]. This means that Lb contributes to maintaining a very low free O_2_ concentration in active symbiotic nodules. In *Medicago truncatula*, the inhibition of N_2_ fixation by adding nitrate to active nodules or in plants grown under constant P deficiencies caused a concerted down-regulation of Lb genes, suggesting a (specific/particular) mechanism capable of lowering the ATP consumption driving nitrogenase [26,28]. These results are in agreement with those of Ott et al. [97], who found that interference of the RNA expression (RNAi) of Lb in nodules of *L. japonicus* resulted in lower ATP/ADP ratios compared with wild-type plants. The existence of an ODB and the presence of Lb for transporting O_2_ to the cytochrome of bacteroids guarantee a minimal free O_2_ concentration, as well as the high rate of respiration required for efficient N_2_ fixation.

## 6. Oxidative Stress

Reactive oxygen species (ROS) are mainly produced by a partial reduction in O_2_ during respiration, since O_2_^−^ radicals and H_2_O_2_ are the most important ROS compounds (1–3% of O_2_ is reduced to ROS) [11]. It has been proposed that the production of H_2_O_2_ is linked to infection and the nodule developmental program [100,101]. Recent results have shown that in nodules of *Phaseolus vulgaris*, the overexpression of Rboh (respiratory burst oxidase homolog responsible for ROS generation, see Puppo et al. [102]) enhanced nodule activity, nodule biomass, and the size and density of bacteroids in symbiosomes [103]. The production of ROS also seems to play an important role in nodule senescence [11]. In indeterminate nodules of pea and alfalfa, Rubio et al. [101] concluded that there was an accumulation of H_2_O_2_ in zone IV (senescent zone), which could be responsible for the loss of bacteroid structural integrity and the oxidative degradation of leghemoglobins (Lb), among other processes linked to oxidative stress. Furthermore, data in the literature has supported the possibility that reactive oxygen and nitrogen species (ROS and RNS) are implied, through the activity of H_2_O_2_ and nitric oxide (NO), in the signaling transduction that regulates nodule activity, by either interacting with Lb or by reducing sucrose synthase activity [104].

Evidence has shown that NO acts as a multi-faceted regulator in the early stages of nodule development and in the senescence program of mature nodules [105]. In parallel, gene expression analysis has indicated that NO is involved in plant defense repression, therefore facilitating optimal plant-microbe interactions and successful nodule formation [106]. In active nodules, NO accumulates in the active zone, triggering senescence processes and reducing N_2_ fixation [106]. Nitrogenase activity is most likely regulated by NO as a consequence of *S*-nitrosylation of the protein, as suggested by predictions using computational models [107]. In addition, Melo et al. [108] found NO to be involved in the regulation of N metabolism in root nodules of *M. truncatula* through an inactivation of GS. They proposed that NO induces an inactivation of cytosolic GS in a post-translational manner through tyrosine (Tyr) nitration [108]. Furthermore, GS inhibition is related to plant defense, and can induce nodule senescence, as well as foster a loss of nodule identity [42]. ROS and NO are tightly related, and these molecules orchestrate the nodule´s developmental processes, particularly in the establishment of symbiosis, linking ROS/NO production to a redox-based regulation of the symbiotic process, in which *S*-sulfenylated and *S*-nitrosylated proteins play an important role [105]. In accordance with this, 20 proteins from *Sinorhizobium meliloti* in symbiosis with *M. truncatula*, including some proteins directly involved in N_2_ fixation, were identified as sulfenylated, suggesting that sulfenylation may regulate the activity of proteins playing major roles in the development and functioning of this symbiotic interaction [109].

## 7. Molecular Control of N_2_ Fixation

Another mechanism controlling the amount of N_2_ fixed by plants is the adjustment of the number of nodules to match the plant N demand. One of the principal negative regulatory mechanisms for nodule formation is the so called autoregulation of nodulation (AON) [110,111]. The CLE (CLAVATA3/endosperm surrounding region-related) peptides is the most thoroughly studied signaling peptide family in plants [36]. Research evidence has shown that nodulation-suppressing CLE peptides are key compounds of the AON system, which are synthesized in roots and induced by transcription factors (e.g., nodule inception (NIN), induced by the Nod-factors produced by bacteria) [112]. Nodulation-suppressing CLE peptides are post-translationally modified and predicted to be glycosylated before travelling to the shoot for binding and activating a Leucine-rich repeat (LRR) receptor-like kinase [113]. The mutation of this receptor-like kinase results in hypernodulation [112]. Afterwards, the activated LRR receptor-like kinase induces the production of a shoot-derived signal that has recently been defined as a microRNA (miR2111), which leads to a down-regulation of the expression of *Too Much Love* (TML), a root active Kelch-repeat containing an F-box protein, which in turn regulates the expression of NIN transcription factors [112,114,115]. Depending on the amount of miR2111 expressed, the production of nodules can be stimulated or repressed (i.e., more mi2111 promotes nodule development). The expression of several peptides of the CLE family in diverse legumes is coupled with the nodule developmental program, including rhizobia-inducted LjCLE-RS1, LjCLE-RS2, LjCLE-RS3, and LjCLE40 [116] or in soybean GmRIC1 and GmRIC2 [117]. Furthermore, CLE peptides play an important role in nutrient responses and can also be induced in uninoculated roots of legumes by nitrate [116,118]. For instance, in *Lotus japonicus*, CLE peptides LjCLE-RS2, LjCLE-RS3, and LjCLE40 were induced by the presence of nitrate; a similar situation has also been described for GmNIC1 [116,117]. However, NIC1, probably locally, rather than systemically, regulates nodule formation [117]. As previously mentioned, nitrate is well-known to inhibit nodule formation, and some evidence has shown that AON and nitrate-nodule inhibition could have some similarities [112]. Recently, the NITRATE UNRESPONSIVE SYMBIOSIS 1 (NRSYM1) gene, encoding a NIN-LIKE PROTEIN transcription factor, was identified as a key regulator in the nitrate inhibition of nodulation in *Lotus japonicus* [119]. This transcription factor regulates the production of CLE-RS2 in response to the presence of nitrate, which, in turn, negatively regulates the nodule number.

The availability of high-throughput and cost effective next generation sequencing (NGS) platforms, as well as high-throughput genotyping technologies in conjunction with the use of bioinformatics, has facilitated the generation of a massive amount of genomic data for model and crop legumes, which are very valuable for studying and understanding nodule regulatory mechanisms, from symbiotic tissue to the whole plant [120,121]. These last-generation functional genomic tools presently constitute one of the most important technological trends that ultimately point to the breeding of more efficient legume cultivars grown under stressful conditions [1]. Similarly, transcriptomics/gene expression studies, using a range of platforms, have been very valuable in the study of nodulated plants grown under stressful environments. These studies have revealed a significant consistency with previously reported physiological studies, generating new discoveries associated with differential gene expression and gene functions [26,110,122,123]. In this sense, split-root experiments with nodulated *Medicago truncatula* plants have shown that N acquisition is controlled by systemic regulation dependent on the N status of the plant, in which a complex gene regulation network between the nodule development program and the N assimilation process is concerned [124]. A study conducted in *M. truncatula* by Cabeza et al. [26] showed that the nodule H_2_ evolution started to decline after about 4 h of NO_3_^−^ application, when a marked shift in nodule gene expression occurred (1120 differentially expressed genes), and the down-regulation of 127 genes for NCR peptides and various nodulins was notable, particularly all the genes of leghemoglobines (Lb). NCR peptides are responsible for bacterial differentiation in bacteroids in legumes of the inverted repeat-lacking clade (IRLC) by the recognition of peptides through the protein BacA (bacteroid development factor A), located in the symbiosome membrane (Figure 1) [125,126].

Phosphorous (P) deficiency constitutes a constraint for N_2_ fixation in legumes, particularly in soils with low P availability [127], which has been associated with slow plant growth and decreases in N_2_ fixation [128]. In low-P soils, legumes depending on N_2_ fixation have responded positively to P fertilization and shown an increased N content in shoots and roots [129]. In addition, P-limited grain legumes have been able to support normal N_2_ fixation for as long as three weeks, exclusively on the basis of seed P reserves [130]. Nodules have proven to be the preferential P sink, showing very high concentrations of P compared to various plant organs. When legumes have been grown under a continuously low P supply, they have mainly allocated the limited P to nodules, while leaves have been strongly depleted before the nodule P concentration has been significantly affected [39]. In turn, when heavily depleted plants have been re-supplied with a limited amount of P, nodules have rapidly reached sufficient P concentrations [131]. Comparative transcriptome and proteome studies have shown complex internal reactions and acclimations of plant organs to low P concentrations [132,133], including long-distance signaling processes involving miRNA and sugars as signal carriers [134]. In *M. truncatula*, the N_2_ fixation activity in plants grown under P-depletion diverged from that of fully nourished plants, primarily because fewer nodules were formed in the P-depleted plants, while the activity of the existing nodules was maintained for as long as two weeks under P shortage [123]. In this study, RNAseq exhibited nodule acclimation with a total of 1140 differentially expressed genes, some of which were upregulated (i.e., genes for P remobilization form organic structures and nodule malate synthesis), while others were downregulated (fermentation genes). It was concluded that plants maintain N_2_ fixation and viable leaf tissue as long as possible during whole-plant P shortages in order to retain their ability to react in case of emerging new P sources. More recently, one low symbiotic efficiency *Mesorhizobium*-chickpea association (MmSWRI9) grown under low P availability showed low P levels in nodules and exhibited the upregulation of several key P starvation-responsive genes, as well as an accumulation of ASN in nodules [135]. Additionally, the levels of identified AAs in P-deficient leaves of MmSWRI9-inoculated plants exceeded the shoot N requirement during P starvation, indicative of N feedback inhibition [135]. Conversely, in this study, P levels increased in nodules of P-stressed McCP-31-incoculated plants (a second *Mesorhizobium*-chickpea association, with a higher symbiotic efficiency under low P than MmSWRI9) because these plants evolved various metabolic and biochemical strategies to maintain nodular P homeostasis under P deficiency. These adaptations included an improved production and exudation of organic acids from the roots into the rhizosphere, the capacity to protect nodule metabolism against P shortage-induced oxidative stress, and the activation of alternative pathways of C metabolism relying on the reprogramming of whole-plant metabolism. In addition, one recent study showed an increase in a metabolic bypass that acted at the PEP branch point in the glycolysis of *Virgilia derivacata* under P starvation [136]. In this work, there were improved activities of nodule PEPC, malate dehydrogenase, and malic enzyme, whereas pyruvate kinase diminished, indicating that an adenylate bypass occurred under P shortage, either to mediate pyruvate via a non-adenylate-requiring metabolic pathway or to synthesize more organic acids.

With respect to the role of hormones like cytokinins in the autoregulation of nodulation (AON), several works have shown that small RNAs are involved in this signal mechanism of nodule formation [114,137,138]. The miRNA, a 21–24 nucleotide RNA product of a non-protein-coding gene, has been observed as playing a pivotal role as signaling molecules [139,140]. A rapidly increasing number of different miRNAs have been reported, in particular from model plants and especially in *Arabidopsis* [141]. Nutrient-uptake-related processes have been proven to be influenced by shoot-borne miRNAs, most likely because miRNA is an efficient pathway for shoot-root signaling. These small RNAs have been identified as regulating a wide spectrum of genes related to plant defense against pathogens [142]; salt and drought stress [143,144]; and nutrient deficiency responses, e.g., micronutrients, phosphate, and sulfur deprivation [145,146,147]. In the case of phosphate (Pi) deprivation, one of the best characterized miRNA regulation processes in plants, the miR399 has been observed to down-regulate PHO2 mRNA levels, thus encoding a ubiquitin-conjugating enzyme responsible for phosphate transporter degradation [145,148]. This mechanism has been indicated to play a central role in maintaining Pi homeostasis in plants [148]. In addition, miRNAs were identified in pathways related to nitrogen metabolism in *Arabidopsis* [149,150]. In leguminous plants, miRNAs have also been identified as playing an important role in the process of organogenesis in nodules [151,152,153]. The over-expression of miR166 post-transcriptionally in *M. truncatula* has been able to regulate the expression of transcription factors related to nodule development and root architecture, especially those of the family class-III homeodomain-leucine zipper (HD-ZIP III) [152]. Along the same line, other studies have also demonstrated that other miRNAs are specifically involved in the nodule development of *M. truncatula* by interfering with the expression of transcription factors [153,154]. For example, miR167 repressed the expression of a gene encoding an auxin response factor (GmARF8) and the miR167-GmARF8 module interrupted the auxin sensing, triggering the proliferation of nodules [155]. A high level of miR167 produces a similar phenotype as supernumeric nodule mutants defective in LRR receptor kinase. More recently, as has been previously mentioned, miR2111 has been proven to have a central role in the regulation of the amount of nodules, although little is known about the role of miRNAs in the regulation of N_2_ fixation in active nodules, and the function that they play in feedback signaling, which may control nodule activity.

## 8. Conclusions and Future Directions

Nodules are very complex organelle-like structures, containing several processes which operate and interact at distinct levels, including N and C metabolism, oxygen flow through the nodule, oxidative stress, and P levels, among others. These processes are very finely tuned on a whole-plant basis. The CA-PEPC-MDH is a key pathway for C feeding and C skeleton provision for N assimilation of the bacteroid. In addition, malate appears to have a crucial role in many aspects of symbiotic N_2_ fixation regulation, which instead of being limited or continuously driven by one single factor, tends to oscillate around limitation by more than one factor, although the malate pool size appears to be a good candidate for mediating such an oscillatory mechanism. The asparagine (ASN) pool size mimics a “feedback” regulatory effect since its synthesis depletes the malate pool. Such a system might allow immediate excess nitrogenase activity to be prevented, although it does not explain the extensive indirect evidence that some phloem-N-related substances can decrease the nitrogenase activity. How plants sense N-status and how this stimulates the regulatory factors discussed above are key issues for understanding N_2_ fixation regulation and must be further studied in the future. Unfortunately, despite a wealth of experimental data identifying the main bottlenecks of N_2_ fixation regulation, there is no unifying theory that explains all of the aspects regulating N_2_ fixation rates and related N-nutrition of legumes to date. Apart from its scientific value, such an understanding is required to impact several aspects of the process, mainly through legume breeding, in order to have agronomic sustainable benefits. Finally, high-throughput functional genomic tools and `omics´ applications are very valuable for the identification of many genes/transcripts/proteins/metabolites that are good candidates for more accurately dissecting the N_2_ fixation regulatory mechanisms associated with physiological responses to abiotic stresses. In addition, the role of specific miRNAs in the regulation of N_2_ fixation will most likely be further studied in the future. Using the genetic molecular methods currently available for the model plant *M. truncatula*, it is now feasible to clearly unravel and decipher the mechanisms underlying the regulation of N_2_ fixation and characterize the signal-transducing elements associated with it.
